# Enhancement of Antiviral T-Cell Responses by Vitamin C Suggests New Strategies to Improve Manufacturing of Virus-Specific T Cells for Adoptive Immunotherapy

**DOI:** 10.3390/biology11040536

**Published:** 2022-03-30

**Authors:** Miriam Laubert, Agnes Bonifacius, Anna Christina Dragon, Caroline Mangare, Rainer Blasczyk, Jochen Huehn, Britta Eiz-Vesper

**Affiliations:** 1 Institute of Transfusion Medicine and Transplant Engineering, Hannover Medical School, 30625 Hannover, Germany; miriam.laubert@googlemail.com (M.L.); bonifacius.agnes@mh-hannover.de (A.B.); dragon.anna@mh-hannover.de (A.C.D.); caroleunice2000@gmail.com (C.M.); blasczyk.rainer@mh-hannover.de (R.B.); 2 Department Experimental Immunology, Helmholtz Centre for Infection Research, 38124 Braunschweig, Germany; jochen.huehn@helmholtz-hzi.de

**Keywords:** T cells, antiviral immunity, cytomegalovirus, vitamin C, hematopoietic stem cell transplantation, cancer therapy

## Abstract

**Simple Summary:**

Leukemia and lymphoma patients are routinely transplanted with hematopoietic stem cells. Due to the required immunosuppression, bacterial, viral and fungal infections are life-threatening complications after transplantation. In recent years, treatment with virus-specific T cells isolated from stem cell, family or third-party donors has emerged as an alternative to conventional therapies. Since vitamins are described to influence the immune system and its cellular components, the aim of this study was to examine whether vitamins modulate virus-specific T-cell function and thereby enable an improvement of therapy. We were able to show that vitamin C increases the expansion and activation state of virus-specific T cells, and an increased influence of vitamin C was observed on cells isolated from male donors and donors above 40 years of age. In conclusion, this study provides insights into the impact of vitamin C on virus-specific T cells, thereby suggesting its potential application as additional selection criteria and a strategy to improve virus-specific T-cell therapy.

**Abstract:**

Allogeneic and autologous transplantation of hematopoietic stem cells (HSCT) are being routinely used to treat patients with leukemia and lymphoma. Due to the required immunosuppression after stem cell transplantation, infection and reactivation by viruses are life-threatening complications. In recent years, adoptive transfer using virus-specific T cells (VSTs) has emerged as alternative to conventional therapies. Since vitamins are described to influence the immune system and its cellular components, the aim of this study was to examine whether vitamins modulate VST function and thereby enable an improvement of therapy. For that, we investigated the impact of vitamin C and D on the functionality of cytomegalovirus (CMV)-specific T cells isolated from CMV-seropositive healthy donors. We were able to show that vitamin C increases the expansion and activation state of CMV-specific T cells, and an increased influence of vitamin C was observed on cells isolated from male donors and donors above 40 years of age. A higher frequency of the terminally differentiated effector memory CD8^+^ T-cell population in these donors indicates a connection between these cells and the enhanced response to vitamin C. Thus, here we provide insights into the impact of vitamin C on cytotoxic T cells as well as possible additional selection criteria and strategies to improve VST functionality.

## 1. Introduction

Hematopoietic stem cell transplantation (HSCT) with allogeneic or autologous stem cells is indicated for a variety of hematological cancers, such as leukemia or multiple myeloma, but also for non-malignant diseases of the blood system or bone marrow [1]. Next to primary disease relapse and graft-versus-host-disease (GvHD) [2], viral infections are one of the most serious complications after HSCT [3]. Infections with or reactivation of human cytomegalovirus (CMV), Epstein-Barr virus (EBV) and adenovirus (ADV) are frequent [4,5,6]. Those infections develop in response to a severely weakened immune system, in particular caused by the dysfunctional T-cell response, resulting from required immunosuppressive therapy. Existing treatment options, such as reduction of immunosuppression, chemotherapy or antiviral medication are associated with severe side effects and drug resistance [7,8,9]. Therefore, other therapeutic concepts were established over the years, whereby one of the most promising approaches is the adoptive transfer of virus-specific T cells (VST) from seropositive donors. In this strategy, VSTs are administered to patients to reconstitute a sufficient antiviral immune response. Various methods to generate VSTs for clinical use have been established and described, such as expansion using transduced antigen presenting cells (APC), stimulation with EBV-transformed B cells, selection by multimers or the cytokine capture system approach after stimulation with viral peptides or peptide pools [10,11,12]. The latter technique offers a rapid manufacturing of VSTs and is thus a promising approach for fast treatment of high-risk patients with poor prognoses [13]. However, all described methods are often limited by donor availability, low T-cell frequencies, impaired T-cell functionality or the first two in addition to long expansion times emphasizing the aim to optimize the generation process.

As previously described, the cellular components of the immune system are influenced by vitamins. Therefore, the influence of vitamins on the phenotype, the proliferation capacity and the functionality of antigen-specific T cells were the focus of this work. Vitamin A has been described as being required for cell differentiation and normal immune function [14]. Resulting in impaired functionality of the immune system, vitamin A deficiency is one of the most common causes of immunosuppression, affecting one-third of children worldwide [15]. Retinoic acid, one of the active forms of vitamin A, is associated with mucosal immunity and tolerance [14]. B vitamins are a class of eight chemically distinct molecules that are coenzymes or precursors and involved in a variety of metabolic processes [16]. Ascorbic acid, better known as vitamin C, is an essential micronutrient for the human immune system. It accumulates in skin cells, stabilizes collagen and enhances wound healing [17]. In skin cells as well as immune cells such as neutrophils and macrophages, vitamin C is taken up by sodium-dependent vitamin C transporter 1/2 (SVCT1/2), suggesting an important role of vitamin C in those cells [18,19]. It is known that vitamin C promotes leukocyte chemotaxis and reactive oxygen species (ROS) generation during phagocytosis and enhances apoptosis [17]. Furthermore, Tan et al. showed that vitamin C decreases the expression of nuclear factor ‘kappa-light-chain-enhancer’ of activated B cells (NFκB) in dendritic cells (DCs) [20], leading to increased development of type 2 T helper cells (Th2) and production of interleukin (IL)-10 in allogeneic T cells when co-cultured with the vitamin C-treated DCs. Little is known about the influence of vitamins on lymphocytes, but it is known that vitamin C accumulates via SVCT and sodium-independent glucose transporters (GLUT) [21]. Moreover, vitamin C enhances differentiation and proliferation of T cells [22,23,24]. Huijskens et al. discovered that, after 3 weeks, vitamin C improved T-cell maturation from hemapoietic stem cells to double-positive T cells in a feeder-free system. Besides, vitamin C is considered to shift T helper cells from Th2 to Th1 and induce their effector cytokines, interferon gamma (IFN-γ) and tumor necrosis factor alpha (TNF-α) [25]. Song et al. suggested that vitamin C affects histone demethylase activity, which increases IL-17 effector cytokine secretion by Th17 cells [26,27]. Furthermore, vitamin C was shown to promote stability of Foxp3 expression in induced regulatory T cells (Tregs) [28,29,30,31]. The effects of vitamin C on different CD4^+^ T-cell subsets have been described to involve epigenetic regulation of levels of histone modification and DNA methylation [32]. However, the influence on cytotoxic CD8^+^ T cells is not well understood yet.

Vitamin D is another vitamin known for its regulatory effects on the immune system. A study by Palmer et al. described that vitamin D compromised the development of Th17 and Th9 cells from naïve CD4^+^ T cells, but increased the frequency of IL-10 expressing cells [33]. Further, the release of IL-4 and IFN-γ from murine naïve CD4^+^ T cells during polarization was shown to be inhibited by vitamin D [34], while in human peripheral blood mononuclear cells (PBMCs), IL-4 was shown to be enhanced by vitamin D [35]. This suggests that vitamin D plays an important role in immune-mediated diseases caused by Th1 and Th2 cells [35,36]. More recently, it was found that the expression of vitamin D receptors was induced by T-cell receptor (TCR) signaling [37].

The aim of this work was to develop strategies to improve the manufacturing and functionality of VSTs. To this end, we investigated the impact of vitamin C and D on CMV-specific T cells with respect to their generation and functionality in order to potentially improve immunotherapeutic T-cell transfer. Vitamin C increased the expansion and functionality of CMV-specific T cells. This was found to be related to donor age and gender, with stronger effects observed in cells derived from male donors and donors above 40 years of age, thereby adding potential new criteria for VST donor selection and optimization of manufacturing strategies.

## 2. Materials and Methods

### 2.1. Isolation of PBMCs and T Cells

PBMCs were isolated from residual blood samples obtained from healthy platelet donors of the Hannover Medical School (MHH) Institute of Transfusion Medicine and Transplant Engineering. Consent was obtained from all donors, approved by the Ethics Committee of Hannover Medical School (3639–2017) (Supplementary Table S1). CMV-seropositive donors were selected and discontinuous-gradient centrifugation was performed to extract PBMCs from the apheresis blood. Untouched isolation strategies performed with the Pan T cell and CD8^+^ isolation kit (both from Miltenyi Biotec, Bergisch Gladbach, Germany) followed by magnetic cell sorting (MidiMACS Separator, Miltenyi Biotec) were used to receive CD3^+^ and CD8^+^ T cells, according to the instructions of the manufacturer. For naïve T-cell depletion, CD45RA and CD62L immunomagnetic MicroBeads (Miltenyi Biotec) were used as described by Mangare et al. [38] to obtain the following naïve/memory T-cell fractions: CD62L positive fraction (CD62L_PF), consisting of naïve (T_N_, CD45RA^+^ CD62L^+^) and central memory T cells (T_CM_: CD45RA^−^ CD62L^+^); CD62L negative fraction (CD62L_NF), consisting of effector memory cells (T_EM_; CD45RA^−^ CD62L^−^) and late effector memory cells (T_EMRA_; CD45RA^+^ CD62L^−^); CD45RA positive fraction (CD45RA_PF), consisting of T_N_ and T_EMRA_; and CD45RA negative fraction (CD45RA_NF), consisting of T_CM_ and T_EM_. Cell numbers were determined using an improved Neubaer counting-chamber and trypan blue (Sigma Aldrich, St. Louis, MO, USA) to differentiate dead from living cells. Purity of all cell populations was invariably over 90%, as determined by multicolor flow cytometry (FACS Canto II, FACSDiva V8.1.2 software, BD Biosciences, Heidelberg, Germany).

### 2.2. Cell Culture 

Cells were cultured in T-cell medium (TM) consisting of RPMI 1640 medium (Lonza, Basel, Switzerland) supplemented with 10% inactivated human AB serum (c.c.pro, Oberdorla, Germany). A total of 1 × 10^7^ cells per milliliter medium were plated overnight or 5 × 10^6^ cells per 500 µL medium were plated for 3 days in 24-well-plates (Sarstedt, Nümbrecht, Germany). Culture conditions were 37 °C and 5% CO_2_.

### 2.3. Vitamin C and Vitamin D

Vitamin C (L-Ascorbic acid 99%; Sigma Aldrich) solved in sterile water was used for all experiments. Vitamin D (1A,25-Dihydroxyvitamin D3) was solved in sterile water with 1% DMSO (both Sigma Aldrich). The utilized DMSO concentration in the final cell cultures was below 0.1%; hence, it was neglectable.

### 2.4. Expansion of CMV-Specific T Cells with Artificial Antigen Presenting Cell Beads (aAPCs) and Vitamins

aAPCs were used for T cell stimulation to ensure that only direct effects of vitamin C or D on T cells were measured. aAPC beads were generated as previously described [39]. Briefly, M-450 Epoxy beads (Thermo Fisher Scientific, Waltham, MA, USA) were coated with HLA-A*02:01 molecules (DimerX, BD Biosciences) and anti-CD28 monoclonal antibody (mAb; BD Biosciences) and loaded with the HLA*02:01-restricted CMV_pp65_495-503_ peptide (NLVPMVATV, A02pp65p, ProImmune, Oxford, UK). For stimulation, 1 × 10^4^ cells were plated with 1 × 10^4^ aAPCs, with vitamin C and vitamin D supplementation in indicated concentrations in aAPC medium in a 96-well round-bottom plate (BD Biosciences). The aAPC medium contained RPMI 1640 (Lonza, Basel, Switzerland), 1% Sodium pyruvate (Sigma Aldrich), 0.4% MEM-vitamins (100×, Thermo Fisher Scientific, Waltham, MA, USA), 1% non-essential amino acids (100×, Thermo Fisher Scientific) and 5% human AB serum (c.c.pro). Further, 50 U/mL of IL-2 (PeproTech, Hamburg, Germany) were added to the cell suspension. Fresh aAPC medium with IL-2 but without vitamin was added on day 3. After 8 days, the cells were harvested and frequencies of A02pp65p-specific (multimer^+^) cells were measured by flow cytometry. Gates were set based on the light scatter properties of leukocytes and at least 10,000 events were acquired. Multimer^+^ cells were gated based on a clearly defined positive population.

### 2.5. Enrichment of IFN-γ Secreting CMV-Specific T Cells by Cytokine Secretion Assay

A cytokine secretion assay was performed to detect IFN-γ secreting CMV-specific T cells by using the IFN-γ Cytokine Secretion Assay Detection Kit (CSA, Miltenyi Biotec) as described in the manufacturer’s instructions. Approximately 1 × 10^7^ PBMCs were stimulated for 4 h with 1 µg/mL 15-mer overlapping peptide pool covering the whole sequence of CMV phosphoprotein pp65 (pp65pp; Miltenyi Biotec) in the presence or absence of 50 µm vitamin C or 500 nm vitamin D (if not indicated differently), followed by labeling of required IFN-γ secreting CMV-specific T cells with a phycoerythrin (PE)-conjugated IFN-γ detection antibody. Sorting of IFN-γ-secreting cells was accomplished with MiniMACS separation columns (Miltenyi Biotec). Cell numbers were determined as described above and percentages of IFN-γ secreting pp65pp-specific T cells were analyzed by flow cytometry. For detection of dead cells, all samples were stained with 7-amino-actinomycin D (7-AAD, BD Biosciences). To determine CD3^+^ IFN-γ^+^ T cells as well as CD4^+^ IFN-γ^+^ and CD8^+^ IFN-γ^+^ cells, fluorescein-isothiocyanate (FITC) anti-CD3, allophycocyanin (APC) anti-CD8 and allophycocyanin/Cyanin 7 (APC/Cy7) anti-CD45 (all BioLegend, London, Great Britain) were used for cell staining. At least 10,000 events were acquired in the CD45^+^ gate.

### 2.6. Antigen-Specific T-Cell Response Determined by IFN-γ ELISpot Assay

Functionality of the different T-cell fractions was analyzed by an IFN-γ ELISpot assay. Briefly, PBMCs or indicated T-cell populations were isolated (day 0), 50 µM vitamin C was added and the cells were incubated overnight in TM at concentrations as given in figure legends in a 24-well tissue culture plate (Sarstedt) at 37 °C and 5% CO_2_. On day 1, cells were resuspended in fresh medium, counted and seeded at a concentration of 2.5 × 10^5^ PBMCs or 5 × 10^4^ effector T cells cells/well, respectively, in a 96-well plate (pre-coated anti-IFN-γ ELISpot plate; Lophius Biosciences, Regensburg, Germany). Cells were incubated overnight with 50 µM vitamin C and 1 µg/mL pp65pp or 1 µg/mL EBV Consensus (both Miltenyi Biotec). Untreated cells and those treated only with vitamin C served as negative controls. As positive controls, cells were stimulated with 1 µg/mL staphylococcus enterotoxin B (SEB; Sigma Aldrich) diluted in RPMI. IFN-γ secretion was detected after 16 h of incubation at 37 °C and 5% CO_2_ using a biotin-conjugated anti-human IFN-γ antibody (mAb 7-B6-1-biotin, Mabtech, Nacka Strand, Sweden), streptavidin-alkaline phosphatase (Mabtech, Nacka Strand, Sweden) and 5-bromo-4-chloro-3-indolyl phosphate/nitroblue tetrazolium (BCIP/NBT Liquid Substrate, Sigma Aldrich). The samples were analyzed on an ‘AID iSpot Reader System’ with ‘AID EliSpot Software Version 7.0′ and spot counting was performed with ‘AID EliSpot Software Version 8.0. All spot counts were mean values of duplicate wells and expressed as spot-forming unit (SFU) per well per 10^5^ CD3^+^ T cells. The cut-off for positive response was set at a spot count of at least two times higher than the negative control.

### 2.7. Flow Cytometry

Cells of interest were stained with different combinations of the following antibodies: FITC anti-CD3, brilliant violet 510 (BV510) anti-CD4, peridinin chlorophyll (PerCP)-conjugated anti-CD4, APC anti-CD8, APC/Cy7 anti-CD8, APC/Cy7 anti-CD45, PE anti-CD45, PE anti-CD45RO, APC/Cy7 anti-CD62L (all BioLegend) and PE-conjugated HLA-A*02:01/CMVpp65p-specific dextramer (Immudex, Copenhagen, Denmark). Surface staining was performed at room temperature for 20 min in the dark and washed with PBS (Lonza, Vervies, Belgium) with 0.1% human AB serum (c.c. pro). 7-amino-actinomycin D (7-AAD; BD Biosciences) was applied prior to flow cytometric analysis to exclude dead cells. All samples were analyzed by multicolor flow cytometry (FACS Canto II, FACSDiva V8.1.2 software, FlowJo_v10.7.1 software; all BD Biosciences). Gates were set based on the forward scatter versus side scatter properties of lymphocytes. At least 30,000 events were acquired in the CD3^+^ gate.

### 2.8. Intracellular Cytokine Staining

After 3 days of incubation in the presence or absence of vitamin C, 1 × 10^6^ cells per sample were stimulated with peptide pool CMV pp65pp for one hour followed by addition of Brefeldin A (BioLegend) and incubation for another 15 h at 37 °C and 5% CO_2_. Cells were resuspended, washed and extracellularly stained using FITC anti-CD3, APC/Cy7 anti-CD8 and PerCP anti-CD4 (all BioLegend). Subsequently, cells were fixed, permeabilized and intracellularly stained with APC anti-TNF-α (BioLegend) using IntraPrep kit (purchased by Beckman Coulter) following the manufacturer’s instructions. Samples were acquired on a FACS Canto 10c, and gates were set based on the forward scatter versus side scatter properties of lymphocytes. At least 30,000 events were acquired in the CD3^+^ gate. Data were analyzed using FlowJo_v10.7.1 software.

### 2.9. Statistics

All statistical evaluations were carried out with Graph Pad Prism 5 software (GraphPad, San Diego, CA, USA). To calculate significances between two data sets, paired or unpaired t tests for normally distributed results or Wilcoxon signed rank tests were applied. For comparison of more than two groups, Friedman’s test followed by Dunn’s multiple comparison test was used. All significance levels were expressed as *p*-values (* *p* < 0.05; ** *p* < 0.01; *** *p* < 0.001; **** *p* < 0.0001).

## 3. Results

### 3.1. Antigen-Specific T-Cell Response against pp65 Is Enhanced upon Vitamin C and Vitamin D Supplementation

To investigate whether vitamin C and D affect antigen-specific T-cell responses, PBMCs from CMV-seropositive donors were stimulated with an overlapping peptide pool covering the whole sequence of CMV protein pp65 (pp65pp) in the presence of different doses of vitamin C or vitamin D. The release of IFN-γ following stimulation was measured by an IFN-γ ELISpot assay.

**Figure 1:**
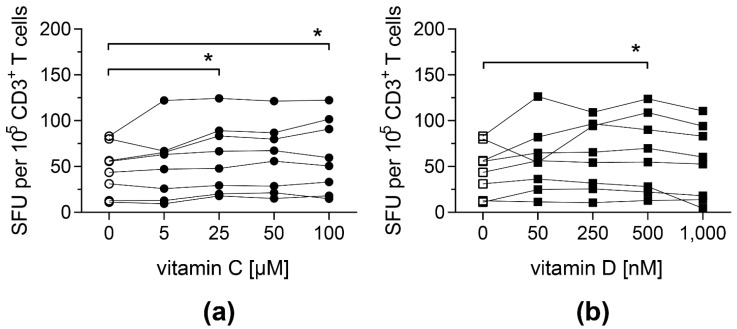
**Frequencies of IFN-γ-secreting T cells upon stimulation with pp65pp are enhanced by vitamin C and vitamin D supplementation.** IFN-γ secretion of T cells was measured by IFN-γ ELISpot assay, in which PBMCs were stimulated with an overlapping peptide pool of the CMV phosphoprotein pp65 (pp65pp) in the presence of the indicated concentrations of (**a**) vitamin C and (**b**) vitamin D. Shown are spot-forming units (SFU) per 10^5^ CD3^+^ T cells. Symbols connected with one line represent data from one donor (*n* = 8). Friedman’s test and post hoc Dunn’s test were used to determine statistical significance (* *p* < 0.05). Only significances to controls not supplemented with vitamins (0 µM) are shown.

Both vitamins led to an improved antigen-specific T-cell response: cells stimulated with pp65pp and supplemented with at least 25 µM vitamin C showed a significantly increased number of cells secreting IFN-γ compared to PBMCs cultured in the absence of vitamin C (Figure 1a). Similarly, when cells were supplemented with 500 nM vitamin D, their IFN-γ secretion was significantly enhanced (Figure 1b). Based on these results, intermediate concentrations of vitamin C (50 µM) and vitamin D (500 nM) were used for further experiments. In addition, T-cell responses towards a peptide pool containing antigens from EBV (EBV consensus) were measured upon addition of vitamin C or D, showing a similar impact of both vitamins (Figure S1).

### 3.2. Vitamin C, but Not Vitamin D, Promotes the Expansion of CMV-Specific Cytotoxic T Cells 

To investigate whether vitamin C and vitamin D increase the proliferative capacity and the functionality of CMV-specific T cells, CD8^+^ T cells from CMV-seropositive, HLA-A*02-positive donors were isolated and stimulated with A02pp65p-loaded aAPCs, IL-2, and vitamin C or D for 7 days. This experimental set-up was chosen to evaluate direct influences of vitamins C and D on T cells and to exclude indirect effects on antigen presentation.

**Figure 2:**
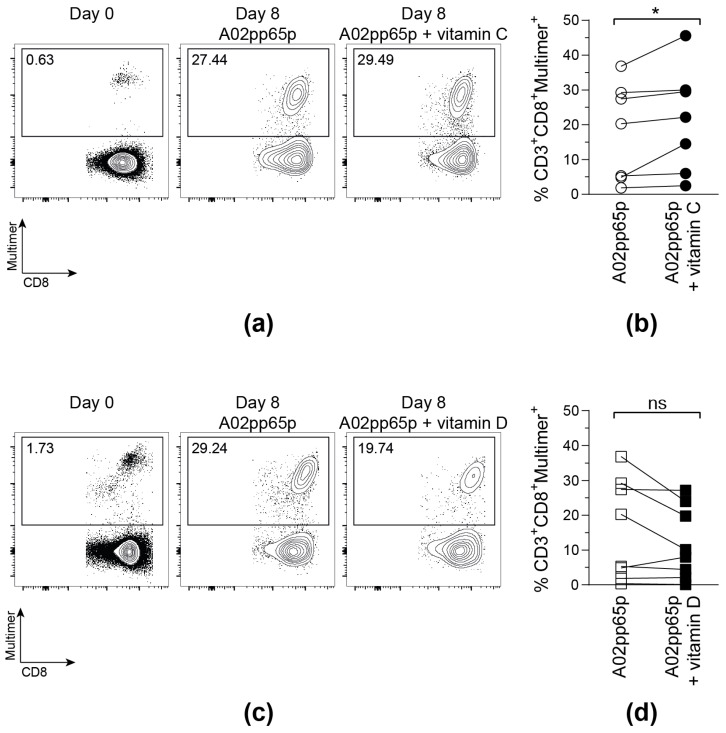
**Vitamin C, but not vitamin D, promotes the expansion of A02pp65p-specific cytotoxic T cells.** Cells are gated on viable CD8^+^ T cells based on scatter properties (FSC-A/SSC-A), CD3 and CD8 expression. Shown are exemplary contour plots (**a**,**c**) as well as summarized frequencies (**b**,**d**) of HLA-A*02/A02pp65p-specific CD8^+^ T cells before (Day 0) and after 8 days (Day 8) of stimulation with A02pp65p-loaded aAPCs. (**a**,**b**) 50 µM vitamin C (A02pp65p + vitamin C); (**c**,**d**) 500 nM vitamin D (A02pp65p + vitamin D) and without addition of vitamins (A02pp65p). Each point ((**b**); *n* = 7), ((**d**); *n* = 8) represents one donor with lines connecting data from the same donor. Friedman’s test and post hoc Dunn’s test were used to determine statistical significance (* *p* < 0.05).

On day 0, all samples contained a low frequency of A02pp65p-specific T cells. Those populations markedly increased after stimulation with A02pp65p-loaded aAPCs for 8 days (Figure 2). Confirming the results obtained via the IFN-γ ELISpot assay, the addition of 50 µM vitamin C led to a significantly enhanced amount of multimer^+^ T cells compared to T cells stimulated in the absence of vitamin C medium. In contrast to that, the frequency of multimer^+^ T cells did not further increase upon vitamin D supplementation, but even tended to be reduced compared to the samples without vitamin supplementation.

### 3.3. The Yield of CMV-Specific T Cells Using a Cytokine Secretion Assay Is Slightly Increased in the Presence of Vitamin C

Supplementation of PBMCs with vitamin C enhanced the frequencies of IFN-γ-secreting T cells upon stimulation with pp65pp as well as the frequency of A02pp65p-specific T cells after expansion in the presence of A02pp65p-loaded aAPCs. Hence, we aimed at determining whether the generation of clinical-grade antigen-specific T cells for adoptive transfer can be improved by the addition of vitamin C. Generation of clinical-grade antigen-specific T-cell products is performed using the cytokine capture system (CCS) IFN-gamma. Here, antigen-specific T cells are restimulated using peptide pools, and IFN-γ-secreting cells are magnetically enriched. The cytokine secretion assay (CSA) is the corresponding laboratory small-scale equivalent to the large-scale clinical-grade process. Therefore, we performed IFN-γ CSA to investigate whether vitamin C supplementation might increase the secretion of IFN-γ and yield of antigen-specific memory T cells during large-scale manufacturing.

**Figure 3:**
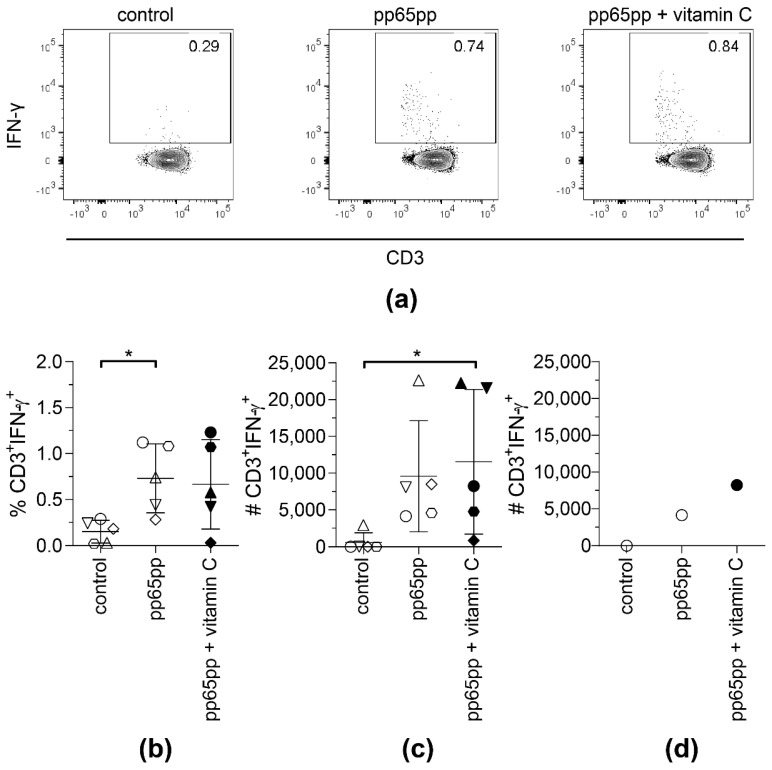
**The yield of CMV-specific T cells using CSA is slightly increased upon supplementation with vitamin C.** CSA was performed using pp65pp and the magnetically enriched cells were analyzed by flow cytometry. Cells are gated on viable CD3^+^ lymphocytes based on CD45 expression, scatter properties (SSC-A), 7-AAD staining and CD3 expression. Number of IFN-γ-expressing T cells after enrichment, without (control), with pp65pp (pp65pp), or with pp65pp and vitamin C supplementation (pp65pp + vitamin C) during CSA shown as (**a**) representative plots, (**b**) frequency of CD3^+^ IFN-γ^+^ T cells and (**c**) absolute numbers of CD3^+^ IFN-γ^+^ T cells (*n* = 5). (**b**,**c**) Each point represents an individual donor and mean ± SD is shown. (**d**) Exemplary number of CD3^+^ IFN-γ^+^ T cells of a 58-year-old male donor. Shown are absolute cell numbers after cell sorting, comparable to results in (**b**). Friedman’s test and post hoc Dunn’s test were used to determine statistical significance (in (**b**,**c**); * *p* < 0.05).

None of the donors showed an increased number of IFN-γ^+^ T cells after cell enrichment and cell sorting when vitamin D was added (data not shown). Therefore, in further experiments, vitamin D was excluded. Vitamin C showed no significant effect on the frequency of IFN-γ-secreting T cells after 4 h of stimulation with pp65pp (Figure 3a,b). However, the absolute number of sorted IFN-γ^+^ T cells supplemented with vitamin C was significantly higher when compared to the controls not supplemented with vitamin C (Figure 3c). Although no significant difference was observed between stimulated cells and stimulated cells with vitamin C supplementation, a difference could be measured for selected donors. Figure 3d shows one representative example where the number of IFN-γ^+^ T cells was enhanced by two-fold when cells were supplemented with vitamin C compared to the respective number of T cells stimulated with pp65pp only. In the same donor, this effect of vitamin C can be seen in the slightly increased percentage of IFN-γ-secreting T cells. Interestingly, samples that benefited from vitamin C supplementation were samples of male donors only.

### 3.4. Antigen-Specific T-Cell Functionality Can Be Improved by Pre-Treatment with Vitamin C for 3 Days

**Figure 4:**
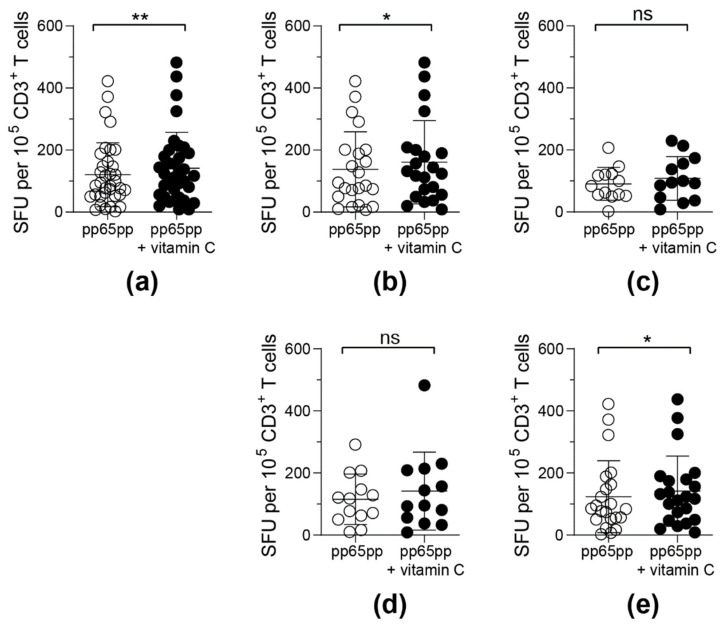
**Frequencies of detectable IFN-γ-secreting antigen-specific T cells upon pre-treatment with vitamin C for 3 days.** PBMCs were incubated with or without 50 µM vitamin C for 3 days followed by activation via pp65pp. By using IFN-γ ELISpot assay, spot forming units (SFU) per 10^5^ T cells were determined. Each symbol represents one donor. ELISpot results obtained were classified into 5 groups: (**a**) all donors (*n* = 35), (**b**) male donors (*n* = 22), (**c**) female donors (*n* = 13), (**d**) donors under the age of 40 years (*n* = 13) and (**e**) donors over the age of 40 years (*n* = 22). Wilcoxon signed rank test was used to calculate statistical significance (* *p* < 0.05, ** *p* < 0.01).

Since the mechanism of how vitamin C affects antiviral T cells is not known, we pre-incubated PBMCS with vitamin C without any antigenic stimulation for 3 days prior to an ELISpot assay in order to observe whether a stronger influence by vitamin C can be detected. After cultivation for 3 days, the amount of CD3^+^ T cells as well as the ratio between CD4^+^ and CD8^+^ T cells did not change significantly upon vitamin C addition (data not shown). Moreover, no influence of vitamin C supplementation on survival and cell numbers was observed (data not shown). On day 3, stimulation with pp65pp was performed and increased numbers of IFN-γ secreting cells in response to sufbsequent pp65pp stimulation were detectable by ELISpot assay (Figure 4a). In addition to the IFN-γ secretion, frequencies of TNF-α-producing CD8^+^ T cells in response to pp65pp were significantly enhanced upon pre-treatment with vitamin C (Figure S2), indicating an overall improved antigen-specific T-cell response.

Upon classification of donors into groups depending on their gender and age, differences in the responsiveness to vitamin C supplementation were detected (Figure 4b–e). T cells obtained from male donors (Figure 4b) and donors above an age of 40 years (Figure 4e) reacted significantly stronger towards pp65pp stimulation after pre-incubation with vitamin C when compared to untreated cells. In contrast, vitamin C pre-stimulation did not affect the functionality of T cells from female donors (Figure 4c) and donors below 40 years of age (Figure 4d).

### 3.5. Vitamin C Does Not Differently Affect Distinct T-Cell Memory Subsets

Based on the results obtained after pre-stimulation of PBMCs with vitamin C prior to antigenic stimulation, we aimed at analyzing whether the impact of vitamin C can be narrowed down to the memory phenotype of the involved T-cell subsets. To this end, flow cytometric analysis of T cells was performed and memory subsets were classified into naïve (T_N_: CD45RA^+^ CD62L^+^), central memory (T_CM_: CD45RA^−^ CD62L^+^), effector memory (T_EM_: CD45RA^−^ CD62L^−^) and late effector memory T cells re-expressing CD45RA (T_EMRA_: CD45RA^+^ CD62L^−^). 

**Figure 5:**
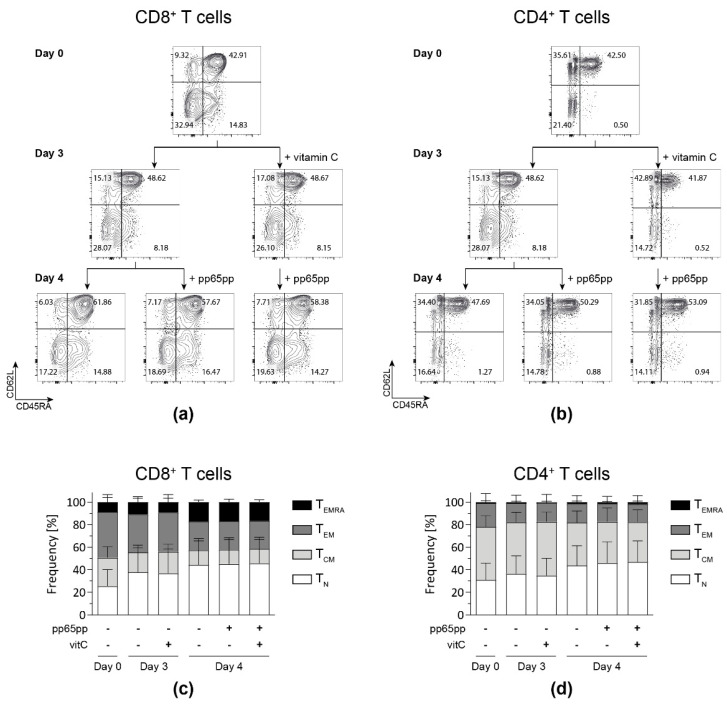
**Vitamin C alone does not affect the memory phenotype distribution yet increases the fraction of T_EMRA_ after antigenic stimulation.** After PBMC isolation, cells were incubated with (+vitamin C) or without vitamin C for 3 days. On day 3, part of the untreated and all treated cells were stimulated overnight with pp65pp. CD45RA and CD62L expression were measured by flow cytometry on days 0, 3, and 4. Cells are gated on viable CD8^+^ and CD4^+^ T cells based on scatter properties (FSC-A/SSC-A), 7-AAD staining as well as CD3, CD8 and CD4 expression. Representative contour plots show percentages of naïve T cells (T_N_; CD45RA^+^ CD62L^+^), central memory cells (T_CM_; CD45RA^−^ CD62L^+^), effector memory cells (T_EM_; CD45RA^−^ CD62L^−^) and late effector memory cells (T_EMRA_; CD45RA^+^ CD62L^−^) among CD8^+^ (**a**,**c**) and CD4^+^ (**b**,**d**) T-cell subsets. Data are shown as representative (**a**,**b**) and summarized results (**c**,**d**) from *n* = 5 donors. Bar graphs represent mean and SD.

Generally, during the time of cultivation, fractions of CD8^+^ and CD4^+^ T_N_ as well as CD8^+^ T_EMRA_ increased, while both CD8^+^ and CD4^+^ T_EM_ fractions decreased (Figure 5). Neither pre-incubation with vitamin C for 3 days nor stimulation with pp65pp affected the distribution of T-cell subsets compared to the respective untreated cells.

### 3.6. Vitamin C Enhances IFN-γ Secretion by CD45RA^+^ T Cells 

In order to assess the influence of vitamin C on the functionality of the different phenotypic subsets of CMV-specific T cells, responses of total PBMCs were compared to cell fractions that were selected based on their CD45RA or CD62L expression. Both of these selection methods were available for the GMP-compliant use in clinical manufacturing and thereby were potentially applicable for VST generation. Functionality of T-cell subsets was assessed by IFN-γ ELISpot assay after pp65pp stimulation and vitamin C supplementation.

**Figure 6:**
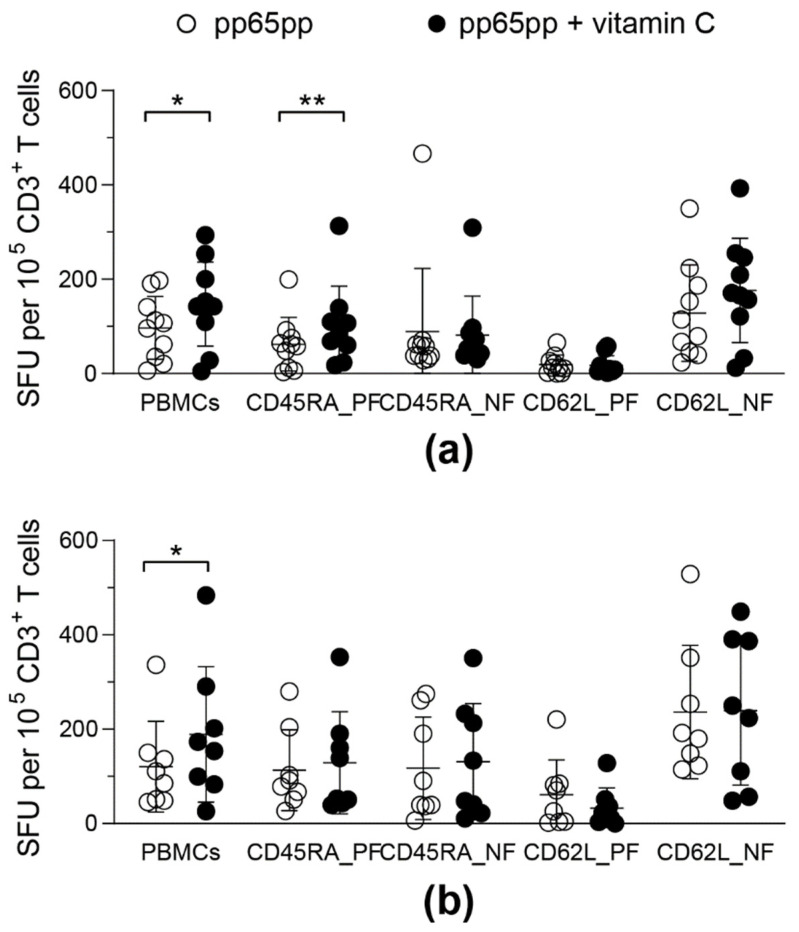
**Vitamin C enhances IFN-γ secretion in CD45RA^+^ T cells.** After PBMC isolation, different T-cell fractions (5 × 10^4^ effector cells) were isolated using CD45RA and CD62L immunomagnetic MicroBeads and collecting the respective positive (PF) and negative fractions (NF). Half of all cell fractions were pre-treated with vitamin C for (**a**) 1 day or (**b**) 3 days. Hereinafter all cell fractions were stimulated with pp65pp overnight. CMV-specific T-cell responses were determined by IFN-γ ELISpot. Shown are spot-forming units (SFU) per 10^5^ CD3^+^ cells after 1 day of incubation with (pp65pp + vitamin C) or without (pp65pp) vitamin C and subsequent stimulation with pp65. Each symbol represents a different donor (*n* = 8–10). Wilcoxon signed rank test was used to determine statistical significance between the same cell fractions; only statistically significant differences are shown (* *p* < 0.05; ** *p* < 0.01).

Isolation of CD45RA positive (CD45RA_PF) and negative fractions (CD45RA_NF) as well as CD62L positive (CD62L_PF) and negative fractions (CD62L_NF) was performed to compare the responses to vitamin C and stimulation with pp65pp in different T-cell subsets. Overall, the IFN-γ ELISpot assay identified higher T-cell responses in the CD62L negative fraction (CD62L_NF), which includes high frequencies of T_EM_ and T_EMRA_. Furthermore, IFN-γ secretion increased after pre-treatment with vitamin C in CD45RA_PF and slightly yet not significantly in CD62L_NF (Figure 6). Both T-cell subsets contain a high frequency of T_EMRA_ (Figure S3). Moreover, the phenotypic analysis revealed that men above an age of 40 years had a slightly higher T_EMRA_ population, while female donors and male donors below 40 years of age had slightly more T_EM_ (Figure S4).

## 4. Discussion

Vitamin C plays an important role in immunologic processes of lymphocytes, which are, however, not yet fully understood [26]. An intake of 100–200 mg vitamin C per day by healthy individuals has been proven to be adequate to saturate the plasma concentrations, which are in the range of 50–80 µM, and were further shown to reduce the risk of chronic disease, such as cardiovascular disease and cancer [40,41,42]. Vitamin C concentrations used in this study were 50 µM, reflecting plasma concentrations. We discovered that vitamin C significantly enhanced antigen-specific T-cell functionality by increasing IFN-γ secretion after stimulation with a peptide pool covering the sequence of the whole CMV phosphoprotein pp65 (pp65pp), particularly after pre-treatment with 50 µM vitamin C for 3 days before stimulation with pp65. A similar effect could be shown by Noh et al. In this study, T-cell cytokines after vitamin C supplementation and sensitization with 2,4-dinitroflourobenzene (DNFB) were analyzed in a murine model. Higher levels of IFN-γ could be observed when vitamin C was subjected simultaneously to sensitization [25]. In the present study, we found that CMV-specific T-cell expansion was increased by vitamin C after antigenic stimulation with artificial antigen presenting cells. This indicates that vitamin C directly acts on CD8^+^ T cells. Since effects of vitamin C were also observed after short-term antigenic stimulation, the higher frequencies of multimer^+^ cells expanded in the presence of vitamin C might not be caused by augmented proliferation. Further mechanisms such as resistance to activation-induced cell death might play a role, e.g., via neutralization of reactive oxygen species (ROS) arising upon activation by vitamin C [43]. More in-depth analyses will be required to elucidate the underlying mechanism. Iamsawat et al. showed that CD4^+^ induced Tregs (iTregs) were stabilized by vitamin C and could thus reduce GvHD after HSCT due to DNA demethylation inducing higher Foxp3 expression [44]. In order to assess if the beneficial effect of vitamin C can be exploited for clinical manufacturing, its impact was evaluated in a CSA, in which the same stimulation and enrichment principle as used in generation of clinical-grade antigen-specific T cells is applied. Upon vitamin C supplementation, an enhanced number of IFN-γ-secreting CD4^+^ and CD8^+^ T cells was yielded by CSA. Since, interestingly, this was more pronounced when using cells isolated from male donors, a more detailed analysis of donor groups was conducted that revealed a differential influence of pre-treatment with vitamin C on the functionality of CMV-specific T cells of donors classified by their gender and age. PBMCs of male donors above an age of 40 years, having slightly larger fractions of T_EMRA_, displayed a higher IFN-γ secretion when treated with vitamin C in contrast to untreated cells. Consistent with that, fractions of either selected CD45RA-positive or CD62L-negative cells, which both contain T_EMRA_ and additionally T_N_ or T_EM_, respectively, showed a high responsiveness towards vitamin C supplementation. In a study by Constantini et al. evaluating the effect of vitamin C supplementation among adolescent competitive swimmers, a significant difference between men and women was found, whereby the duration and severity of colds in men was reduced by half by but there was no effect on women [45]. Moreover, it has been estimated that about 20% of total CD8^+^ T cells are CMV-specific and the frequency is even higher in the elderly due to intermittent antigenic stimulation and subclinical reactivations of the virus throughout the lifetime, resulting in memory inflation with accumulation of T_EMRA_ [46]. Multimer analysis by Appay et al. to detect antiviral T cells showed that 50–60% of pp65-specific T cells were present in the T_EMRA_ population [47], which would explain the increased T-cell response in the CD45RA_PF and CD62L_NF upon vitamin C supplementation among men above 40 years of age.

Overall, our results showed that treatment with vitamin C improves the functionality of CMV-specific T cells, whereby the gender and age of donors influenced the responsiveness towards vitamin C. Those differences in CMV-specific T-cell responses could be attributed to variabilities in memory T-cell subset distribution among donors. Accordingly, the influence of vitamin C on T-cell responses against CMV and other viral infections should be continually studied in larger cohorts to establish dose-response relationships for effective T-cell responses against viral infections and malignancies caused by those. For example, the addition of vitamin C in the clinical manufacturing of VSTs could be beneficial, especially if the selected donor is male or above 40 years old. Vitamin C pre-treatment could potentially lead to a higher yield of VSTs and, moreover, even more functional T cells. With the incorporation of vitamin C supplementation into donor screening and selection strategies, predictions of whether vitamin C supplementation can improve the yield of CD3^+^/IFN-γ^+^ T cells for the production of clinically applicable products will be possible. For this, the overall effects of supplementation still need to be studied in a larger cohort of donors. Based on these data, common characteristics of responsive donors could be identified and then considered for donor selection. Identification of donors responding to vitamin C supplementation could also be supported by lifestyle and dietary surveys of these donors. Based on the results from this study, the impact of vitamin C on T-cell functionality should be investigated in in vivo models of virus-associated malignancies such as prost-transplant lymphoproliferative disorder, an EBV-associated B-cell malignancy. It is also conceivable to conduct a randomized controlled trial with vitamin C supplementation before the donation to compare the functionality of VSTs. Considering phenotypes when choosing a donor could also potentially enhance VST functionality in addition to vitamin C supplementation.

## 5. Conclusions

Together, the results of this study will be of importance particularly for immunosuppressed individuals following allogeneic HSCT with a greater risk of viral infections and reactivation especially by opportunistic herpes viruses, which might result in the development of virus-associated lymphoproliferation and cancer. We were able to show that vitamin C treatment could potentially improve adoptive T-cell transfer in terms of VST yield during manufacturing as well as the effectiveness of VSTs in their defense against viruses. Furthermore, due to the different metabolic requirements of immune cells, investigation of the effects of B vitamins on immune cells is of great interest. Their influence on antiviral T-cell immunity as well as possible synergistic effects of different vitamins should be the subject of future studies.

## Data Availability

All data generated during this study are included in the manuscript.

## Supplementary Materials

The following Supplementary Materials are available online at https://www.mdpi.com/article/10.3390/biology11040536/s1, Figure S1: Frequencies of IFN-γ-secreting T cells upon stimulation with EBV Consensus are enhanced by vitamin C and vitamin D supplementation. Figure S2: Vitamin C enhances TNF-α expression of CD8^+^ T cells. Figure S3: Isolation of CD45RA positive (CD45RA_PF) and negative fractions (CD45RA_NF) as well as CD62L positive (CD62L_PF) and negative fractions (CD62L_NF). Figure S4: Frequencies of T-cell subsets differ in cells obtained from donors categorized by age and gender. Table S1: Number of donors included in each figure, distributed according to age (below or above 40 years of age) and gender.
