# Supplementary material for: Enhancement of Antiviral T-Cell Responses by Vitamin C Suggests New Strategies to Improve Manufacturing of Virus-Specific T Cells for Adoptive Immunotherapy
Source: Biology (Basel). 2022 Mar 30;11(4):536. doi: 10.3390/biology11040536 (PMC9032103; doi:10.3390/biology11040536)
Supplement: Supplementary file 1 [file biology-11-00536-s001.zip › biology-1634217-sup.pdf]

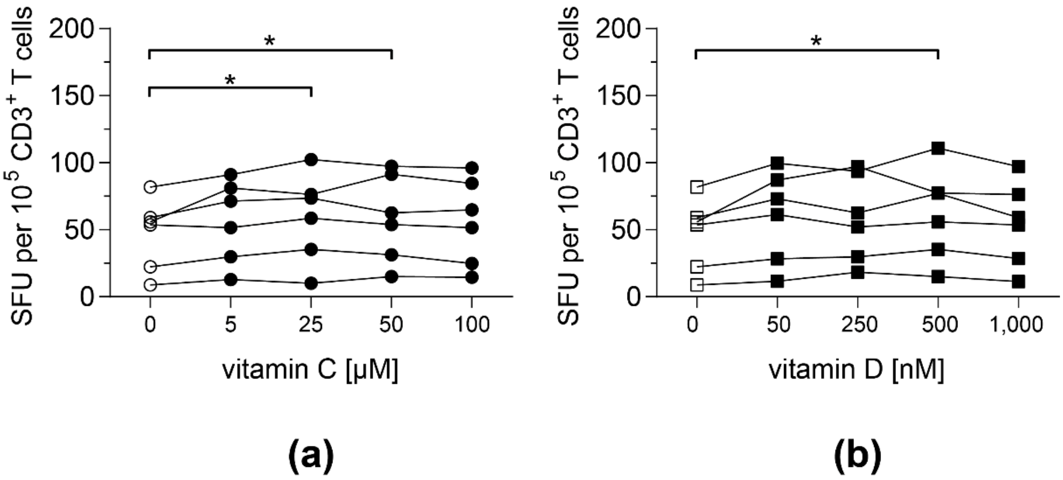


**Figure S1.** Frequencies of IFN-γ-secreting T cells upon stimulation with EBV Consensus are enhanced by vitamin C and vitamin D supplementation. IFN-γ secretion of T cells was measured by IFN-γ ELISpot assay, in which PBMCs were stimulated with a peptide pool containing peptides of EBV in presence of the indicated concentrations of (**a**) vitamin C and (**b**) vitamin D. Shown are spot-forming units (SFU) per $10^5$ $CD3^+$ T cells. Symbols connected with one line represent data from one donor ($n = 6$). Friedman's test and post hoc Dunn's test were used to determine statistical significance (*$p < 0.05$; **$p <$ 0.01). Only significances to controls not supplemented with vitamins (0 μM) are shown.

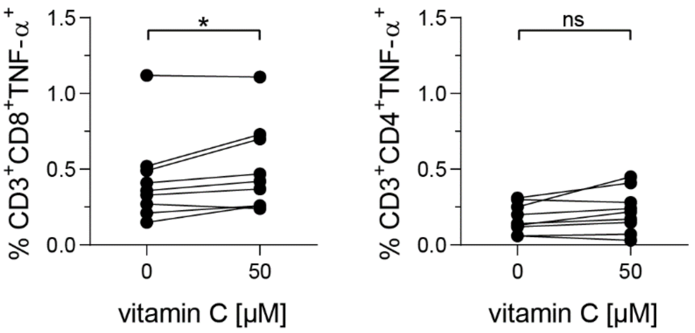


**Figure S2.** Frequencies of TNF-α-expressing $CD8^+$ T cells are enhanced by vitamin C supplementation. After pre-incubation with (50) or without (0) vitamin C for 3 days, T cells were stimulated with peptide pool CMV pp65pp for 12 hours. Intracellular staining was performed and samples were analyzed via flow cytometry. Cells are gated on viable lymphocytes based on scatter properties (FSC-A/SSC-A) and CD3 expression. Shown are frequencies of TNF-$\alpha^+$ cells among $CD8^+$ (left) and $CD4^+$ T cells (right). Symbols connected with one line represent data from one donor ($n = 9$). Statistical analysis was performed using a Wilcoxon signed rank test (ns not significant; *$p < 0.05$).

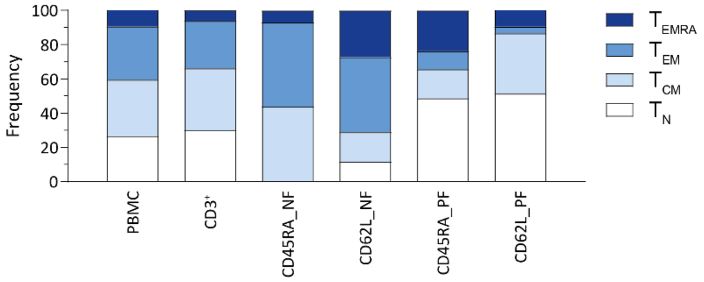


**Figure S3.** Isolation of CD45RA-positive (CD45RA_PF) and -negative fractions (CD45RA_NF) as well as CD62L-positive (CD62L_PF) and -negative fractions (CD62L_NF). $CD3^+$ T cells were isolated from PBMCs of healthy donors and the different fractions were collected from the respective column flow-through and eluated using CD45RA and CD62L MicroBeads. Immunophenotypic analysis was performed by flow cytometry. Cells are gated on viable singlet $CD3^+$ T cells based on scatter properties (FSC-A/FSC-H; FSC-A/SSC-A), CD45 and CD3 expression. Shown are frequencies of the T-cell memory subsets: naïve ($T_N$; $CD45RA^+$ $CD62L^+$), central memory (TCM; $CD45RA^-$ $CD62L^+$), effector memory ($T_{EM}$; $CD45RA^-$ $CD62L^-$) and late effector memory T cells ($T_{EMRA}$; $CD45RA^+$ $CD62L^-$) within PBMCs, $CD3^+$ cells, CD45RA_NF, CD62L_NF, CD45RA_PF and CD62L_PF.

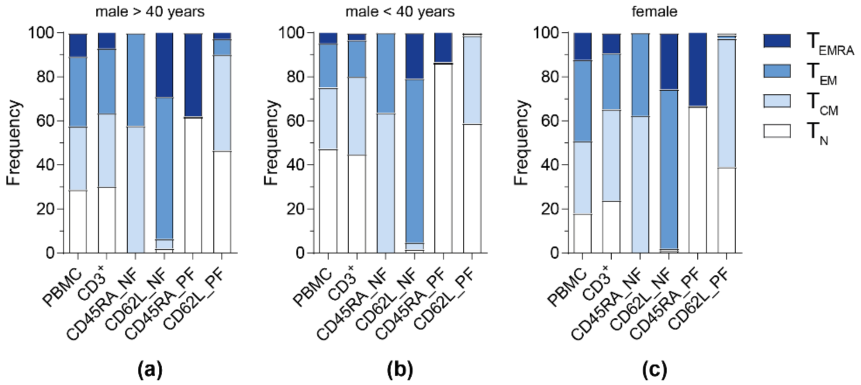


**Figure S4**. Frequencies of T-cell subsets differ in cells obtained from donors categorized by age and gender**.** After naïve T-cell depletion using CD45RA and CD62L MicroBeads, T-cell subsets were divided in the following groups: (**a**) T-cell phenotypes within the different T-cell fractions among males above the age of 40 (**b**) T-cell phenotypes within the different T-cell fractions among males below the age of 40 (**c**) T-cell phenotypes within the different T-cell fractions among females. Cells are gated on viable singlet $CD3^+$ T cells based on scatter properties (FSC-A/FSC-H; FSC-A/SSC-A), CD45 and CD3 expression. PF: positive fraction, NF: negative fraction, $T_N$: naïve T cell ($CD45RA^+$ $CD62L^+$), $T_{CM}$: central memory T cell ($CD45RA^-$ $CD62L^+$), $T_{EM}$: effector memory T cell ($CD45RA^-$ $CD62L^-$), $T_{EMRA}$: late effector memory T cell ($CD45RA^+$ $CD62L^-$).

**Table S1.** Number of donors included in each figure, distributed according to age (below or above 40 years of age) and gender.

| **Figure** | **1** | | **2** | | **3** | | **4** | | | | | **5** | | **6** | |
|---|---|---|---|---|---|---|---|---|---|---|---|---|---|---|---|
| **Panel** | **a** | **b** | **b** | **d** | **b** | **c** | **a** | **b** | **c** | **d** | **e** | **c** | **d** | **a** | **b** |
| total (n) | 8 | 8 | 7 | 8 | 5 | 5 | 35 | 22 | 13 | 13 | 22 | 5 | 5 | 10 | 8 |
| m (n) | 6 | 6 | 6 | 6 | 4 | 4 | 22 | 22 | - | 8 | 14 | 3 | 3 | 7 | 5 |
| m<40 (n) | 2 | 2 | 1 | 1 | 1 | 1 | 8 | 8 | - | 8 | - | 2 | 2 | 2 | 1 |
| m>40 (n) | 4 | 4 | 5 | 5 | 3 | 3 | 14 | 14 | - | - | 14 | 1 | 1 | 5 | 4 |

| | | | | | | | | | | | | | | | |
|---|---|---|---|---|---|---|---|---|---|---|---|---|---|---|---|
| f (n) | 2 | 2 | 1 | 2 | 1 | 1 | 13 | - | 13 | 5 | 8 | 2 | 2 | 3 | 3 |
| f<40 (n) | 1 | 1 | 1 | 1 | - | - | 5 | - | 5 | 5 | - | - | - | 2 | 2 |
| f>40 (n) | 1 | 1 | - | 1 | 1 | 1 | 8 | - | 8 | - | 8 | 2 | 2 | 1 | 1 |

m: male; m < 40: male below 40 years of age; m > 40: male above 40 years of age; f: female; f < 40: female below 40 years of age; f > 40: female above 40 years of age.
